# Identifying Bacteria with Public Health Significance from Farmed Nile Tilapia (*Oreochromis niloticus*), Zambia

**DOI:** 10.1155/2023/6650378

**Published:** 2023-06-09

**Authors:** Bertha Chitambo, Musso Munyeme, Bernard Hang'ombe

**Affiliations:** ^1^Department of Paraclinical Studies, University of Zambia School of Veterinary Medicine, Lusaka, Zambia; ^2^Department of Disease Control, University of Zambia School of Veterinary Medicine, Lusaka, Zambia

## Abstract

Zambia has seen rapid development in aquaculture, and in recent years, the industry has experienced disease outbreaks where fish have increasingly become a potential contributor to emerging bacterial zoonotic diseases. The aim of this study was to identify bacterial pathogens with zoonotic potential in apparently healthy fish and water from their habitat. A total of sixty-three fish were sampled, and fifty-nine water samples were collected from the habitats of these fish. Bacteria were cultured from the internal organs of fish and water, and these were identified through standard bacteriological methods comprising morphological characterization, Gram-staining, and a panel of biochemical tests. The following bacterial pathogens with zoonotic potential were identified at a farm prevalence of *Aeromonas* (13.2%), *Bacillus* (2.1%), *Clostridium* (2.1%), *Escherichia coli* (0.7%), *Klebsiella* (6.9%), *Lactococcus* (2.1%), *Listeria* (0.7%), *Staphylococcus* (18.1%), and *Streptococcus* (0.7). Other bacteria with varying significance as fish pathogens identified included *Acinetobacter* (2.1%), *Aequorivita* (1.4%), *Aerococcus* (1.4%), *Bordetella* (2.1%), *Carnobacterium* (10.4%), *Citrobacter* (3.5%), *Corynebacterium* (1.4%), *Dermatophilus* (1.4%), *Enterococcus* (2.1%), *Flavobacterium* (4.2%), *Micrococcus* (6.9%), *Planococcus* (1.4%), *Proteus* (1.4%), *Pseudomonas* (6.3%), *Rhodococcus* (1.4%), *Shewanella* (1.4%), *Streptococcus* (0.7%), and *Vagococcus* (0.7%). The current study provides baseline information for future reference and the implementation of public health guidelines with regard to potential zoonotic diseases in fish.

## 1. Introduction

Fish is the most affordable protein for many people in densely populated countries, including Zambia [1]. Increased demand for fish protein with escalating population growth has led to rapid growth in aquaculture farming in Zambia and in other countries worldwide [1–3]. Rapid aquaculture development in Zambia has created a potential danger of predisposing fish consumers and workers on fish farms and those in fish processing plants to fish zoonotic disease outbreaks (FZDOs). Worldwide, about 3 billion people derive almost 20 percent of their average per capita intake of animal protein from fish [4]. Fish nutrition is an important source of energy and protein and provides a range of essential nutrients and vitamins to many households worldwide.

The possibility of the emergence of risk factors for FZDOs through handling or ingestion of fish and fish products is ever-increasing [5]. There is a well-documented group of pathogens indigenous to the aquatic environments which have been associated with FZDOs. These pathogens have been isolated from open wounds in highly exposed fishermen and fish handlers [6]. The principal zoonotic fish pathogens of concern are *Aeromonas hydrophila*, *Edwardsiella tarda*, *Mycobacterium marinum*, *Streptococcus iniae*, *Vibrio vulnificus*, and *Vibrio damsel* [7]. *Mycobacterium species*, *Streptococcus iniae*, *Clostridium botulinum*, and *Vibrio vulnificus* are of particular zoonotic importance and concern [8]. Intensive and confined fish rearing in aquaculture predisposes fish to a higher risk of bacterial load on their external surfaces, and contaminated fish are therefore more likely to transmit the infection to humans [5]. Additionally, FZDOs are often related to management factors, such as the quality and quantity of nutrients in the water and high stocking densities, which can increase the bacterial load on the external surface of fish.

This study was conducted on three fish farms located in Chirundu district of Lusaka Province and Siavonga district of Southern Province. The purpose of this study was to identify bacteria with zoonotic potential from fish and water. The bacteria were collected from apparently healthy fish and water and identified by standard bacteriological methods comprising morphological characterization, Gram-staining, and a panel of biochemical tests. The farms involved in this study were assessed to ascertain their health status and whether there were any disease outbreaks.

## 2. Materials and Methods

### 2.1. Study Area

**Figure 1:**
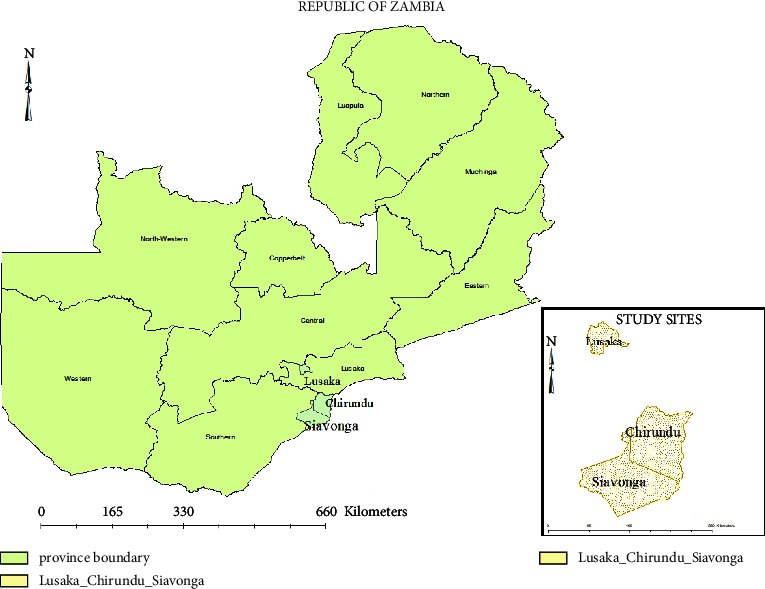
Map of the study area. The study areas included two districts, namely, Chirundu (16.0271°S 28.8509°E) and Siavonga (16.5323°S, 28.7111°E).

The study was conducted in Lusaka and the Southern Province of Zambia. The study areas were selected based on the number of commercial fish production activities. The study areas included two districts: Chirundu (16.0271°S, 28.8509°E) and Siavonga (16.5323°S, 28.7111°E (Figure 1).

### 2.2. Study Design and Sampling

A cross-sectional study was carried out during September and December 2020 to investigate bacteria of zoonotic potential from healthy fish and their environment (water). Healthy fish ranging from fingerlings to out-growers with varying weights ranging from 1 gram to 600 grams from farms designated A, B, and C, reared in ponds and cages were purposively sampled. The fish were caught by dip-netting, sacrificed by stunning to the head, and put in sterile packs. Water samples were collected from the respective ponds and cages of these fish and from Kafue River (water source for farm A) and Kariba dam (water source for farms B and C) in sterile 100 ml sterile bottles. A total of 44 samples each from farms A (15 fish/29 water) and C (29 fish/15 water) were collected, and 34 samples (19 fish/15 water) from farm B (63 fish and 59 water samples for the whole study) were collected. The collected fish and water collected were transported at 4°C to the University of Zambia, School of Veterinary Medicine (UNZA) in Lusaka for further analysis. From each fish, the following organs were collected: gills, liver, spleen, and intestines. From each of these organs, swabs were collected for bacteriology during postmortem. Swabs from sampled water after being centrifuged at 2,500 rpm for 5 minutes were also collected.

Data regarding exposure factor locations in this study were collected with on-farm visits to observe the farming sites and operations as well as the surrounding environments. Through this, the exposure factor locations were determined (Table 1).

### 2.3. Bacterial Isolation

Bacterial swabs collected from fish organs and water were aseptically streaked on MacConkey agar (HiMedia, India), nutrient agar (NA: HiMedia, India), and blood agar (HiMedia, India). The plates were then incubated at 37°C for 48 hrs, and pure cultures were obtained by subculturing and incubating at 37°C for 48 hrs.

### 2.4. Biochemical Characterization

The isolates were identified by determining colony morphology which included shape, color, pigmentation, hemolytic activity, size, edges, and elevation, and afterwards, the isolates were grouped accordingly. Two to three representative isolates from each group were subjected to Gram-staining [9, 10]. Conventional biochemical tests were then used to characterize the bacteria. A loopfull of bacteria was aseptically added to 5 ml of phenol red broth containing 1% sugar and incubated at 37°C for 24 hrs to test for fermentation of different sugars. Sulphur reduction, indole production, and motility tests were determined by using sulphide indole motility (SIM) media again by adding a loopfull of bacteria and incubating at 37°C for 24 hrs. Further identification of isolates was according to Buller [11] (Table 2).

### 2.5. Data Analysis

**Figure 2:**
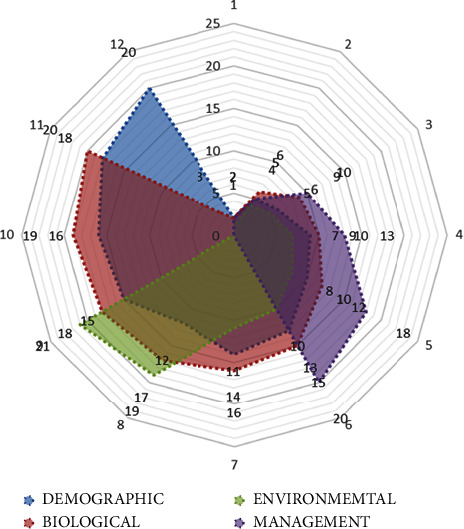
Overlap of the four main descriptive factors within the thematic areas under demographical, biological, environmental, and management factors, with a list of subfactors incorporated within.

The data obtained was entered in Microsoft Excel sheets 2007, cleaned, and exported to Stata SE 12 (https://www.stata.com/) for survey analysis. A descriptive statistical analysis of quantitative bacterial counts and measurements of location was used to describe the outcome of interest. The results were presented in percentages/proportions, and the difference in the distribution of predictor variables was considered significant if the *p* value was less than 0.05 at a 95% confidence interval. Pearson's chi-square was used to test the significance. A spider web analysis was used to analyze the overlap between exposure factors generated under the overall descriptive epidemiology from key thematic areas. Both Table 1 and Figure 2 present basic data based on descriptive epidemiology. Table 1 shows the four main thematic areas: demographical, biological, environmental, and management factors, and each had several descriptors listed under each thematic area (Table 1). Figure 2, on the other hand, used the data from Table 1 to visualize the extent of interaction, overlap, and, to a greater extent, dominance of the factors within these epidemiological descriptors that had a greater influence. Using the spiderweb analysis, the list of factors within each thematic area was collapsed within the data points in the polar or spider chart. Thus, each of the thematic area was grouped together with other existing thematic areas to describe their interrelationship (Table 3).

## 3. Results

A total of 122 samples were collected including fish 51.6% (*n* = 63) and water 48.4% (*n* = 59) from farms A, B and C. The details are shown in Table 2.

A total of 27 bacteria genera were identified. Of particular interest, the following genera and their corresponding prevalence at farm level were found: *Aeromonas* (13.2%), *Bacillus* (2.1%), *Clostridium* (2.1%), *Escherichia coli* (0.7%), *Klebsiella* (6.9%), *Lactococcus* (2.1%), *Listeria* (0.7%), *Staphylococcus* (18.1%), and *Streptococcus* (0.7%). Several other bacteria were isolated as well although some of the bacteria isolated from farms B and C were not properly identified and were presented as ‘unidentified bacteria' (Table 4). There was a significant difference (*p* < 0.0001) in the occurrence of the different bacteria isolated from fish versus water per farm, as shown in Table 2.

The identified exposure factor locations from visual determination of the three farms under study are shown in Table 1. The cage production system comprised the out-grower cage and the juvenile cage, while the other types of production fell under the pond production system.

The four factor location aggregates identified from data collected were as follows: demographical, biological, environmental, and management factors. When the analysis was undertaken, controlling for production types, demographical factor, and location covered a wider area, followed by biological factors locations (Figure 2). Environmental factors and location were closely associated with biological factors, while management factors and locations overlapped across all factors and locations, albeit as an outlier (Figure 2).

## 4. Discussion

The prevalence of bacteria isolates in fish with zoonotic potential reported in this study provides important pre-emptive baseline data as well as an early warning system concerning the formulation and implementation of public health guidelines for the management and control of zoonotic diseases in fish. This present study has identified twenty-seven different genera of bacteria from the apparently healthy tilapia *Oreochromis niloticus*. Some of these isolates are well-known etiological pathogens that cause diseases in both fish and humans. Seong-Joon et al. [12], in a similar study, reported fifteen bacteria genera isolated from eels, three of them *Aeromonas*, *Citrobacter*, and *Pseudomonas* species which have also been identified in this present study. In another study from Tilapia in Trinidad, they also isolated thirteen bacteria genera with five of these genera being *Pseudomonas*, *Staphylococcus*, *Enterobacter*, *Bacillus*, and *Aeromonas* species also reported in our present study [13]. *Aeromonas sobria* and *A. hydrophila* are well-known virulent pathogens of fish worldwide, isolated from clinically sick fish associated with high mortalities [14–16]. Regarding proportional representation, in our current study, *Staphylococcus* spp. had the highest prevalence rate at 18.1%. *Aeromonas* species also had a higher prevalence of 13.2%, a finding similar to reports in Uganda [17] and other parts of the world [12]. The other most common bacteria isolated in this present study included *Klebsiella*, *Pseudomonas*, *Carnobacterium*, *Streptococcus*, and *Lactococcus* spp. The prevalence rates of the other bacteria genera were relatively low.

Although theoretically, streptococcosis affects all sizes of fish [18], bigger fish tend to be more susceptible to other infections such as *Pseudomonas* [19], *Flavobacterium* [20], and other *Streptococcus* species [21–23]. *Aeromonas*, *Staphylococcus*, *Lactococcus*, and *Streptococcus* species are well-known zoonotic bacterial pathogens of fish frequently isolated from diseased fish. The symptoms of fish diseases associated with these bacterial pathogens are skin ulceration, abnormal swimming (swimming in circles), blindness (whitish appearance of the eyes), and exophthalmos. Other authors have also reported similar symptoms with *Aeromonas* species infections in fish [24], *Lactococcus garvieae* [25–27], *Streptococcus iniae* [23, 28], and *Streptococcus agalactiae* [22, 29].

Bacteria from the family streptococcu*s* are well-known opportunistic bacteria in natural aquatic environments. When poor husbandry and excessive stocking density are practiced on a farm, it predisposes the fish to clinical diseases caused by this family of bacteria [27]. Other bacteria species isolated in this study have also been associated with a few cases of disease outbreaks in fish with varying pathogenicity which include *Pseudomonas aeruginosa* [30], *Citrobacter* species [31], and *Klebsiella* species [32]. These bacteria species were isolated from apparently healthy fish and cannot be directly linked to any disease. In this study, no information was readily available on the pathogenicity of *Planococcus* spp., *Shewanella* spp., *Dermatophilus* spp., and *Micrococcus* spp. in fish. The pathogenicity of most bacteria, although ubiquitous in the aquatic environment, depends on stress in the fish host to cause disease [33–35]. Intensive fish farming, high stocking density, and increased human activities in intensive fish farming affect water quality and lead to environmental deterioration that may give rise to the emergence of rare zoonotic bacterial fish diseases in the future [17]. This hypothesis is supported by having isolated the following well-documented zoonotic bacteria: Species in fish: *Aeromonas*, *Klebsiella, Bacillus* [36, 37], *Staphylococcus*, *Listeria* [37]*, Clostridium* [20, 38], *E. coli* [39], *Lactococcus*, and *Streptococcus*. Humans get infected through the consumption of raw or undercooked fish, although *Lactococcus*, *Streptococcus*, and *Staphylococcus* spp. can be transmitted through abraded, wounded skin or through injuries caused by fish fins or fish spikes during handling and processing.

Water samples from ponds had a significantly higher prevalence of bacterial contamination (*p* > 0.0001) than water from cages. This difference can partly be associated with the high stocking density of fish in ponds compared to cages which are placed in lakes with fresh running water continuously. Water recirculation, stock movement contamination, and high organic matter deposition are more rampant in the earthen pond production system as compared to the cage production system. Transmission of bacteria from water to fish and/or humans in aquaculture systems can also easily be facilitated by the high stocking density of fish and by direct contamination of the pond soil lining which may be responsible for the high prevalence and diversity of bacteria observed in earthen ponds as compared to cage production systems. Certain bacterial isolates could not be identified against any of the bacteria profiles used as presented by Buller [11].

In this study, four factors that could give rise to a hazard in food safety were identified: demographical, biological, environmental, and management factors. These factors can occur at various stages of the food chain (farm to table) and involve the contamination of food by different causal agents that can be of biological origin (parasites, viruses, bacteria, fungi, or prions) or chemicals (heavy metals, natural toxins, or organic compounds), risking the health of consumers [40, 41]. Some of the causes of the loss of food safety are inadequate or absent hygiene conditions and practices, high degree of handling, and the use of contaminated water or raw materials [40–42].

The exposure factors that could give rise to hazards in food safety and disease were observed from various locations on the fish farms in this study (Table 1) during on-farm visitations and observations. The results indicated that water source, stocking density, seining practices, control of piscivorous birds, and disposal of dead fish were the main exposure factors to bacterial pathogens in fish/aquatic environments and humans. Other factors included pond preparation and treatment and excess vegetation around ponds for fish farms that used ponds. Farm A would drain their ponds completely dry after the end of the harvesting cycle without removing the mud. The ponds were treated with lime and left to dry out before restocking the fish. This method of pond preparation and treatment, if not done correctly, increases the chances of exposing new fish stocks to infectious pathogens from the previous fish stocks [43].

Water supply and the frequency of changing the water is another important factor. Farms A and C with the pond production system had their water sources from the Kafue river and Kariba dam, respectively. Water was made available to the ponds via dam liners, and this water was treated before filling the ponds. This would at times take longer for the water to be changed at times, leading to compromised water quality due to accumulation of fish fecal material [44]. Poor water quality leads to low dissolved oxygen levels, low pH values, and high nitrate and ammonia levels [45]. Poor water quality is also a result of overstocking. Overstocking either in cages and ponds may also lead to cannibalism behavior in fish due to food competition. When this happens, the skin may break, injure, or get wounded, enhancing pathogen entry. In addition, food competition due to overstocking leads to stunted growth and reduced immunity [43].

Seining practices are also part of exposure factors that lead to the spread of pathogenic factors on fish farms, as observed on the fish farms in our study. Farmers used the same fishing nets between ponds in farms A and C and between cages in farm B between fish harvesting without prior disinfection. The seining equipment may act as a vehicle to transfer infectious agents from one pond/cage to another. A correlation between seining activities and transmission or outbreaks of *Aeromonas hydrophila* in cultured catfish was reported in Alabama, USA [46]. The use of separate equipment in different fish ponds/cages and prior disinfection are recommended.

One of the ways to reduce the chances of an infection spreading on a fish farm is to promptly remove and appropriately dispose of moribund and dead fish. In this study, dead fish were left in ponds/cages for longer before removal or left to be feasted upon by other fish, and this perpetuates the cycle of infection. Cannibalism of infected fish acts as a possible route for the transmission of streptococcal infection in fish. The farmers who used disposal methods buried the dead fish in pits, though at some farms, it was observed that workers would eat the moribund or recently dead fish. Piscivorous birds can also feast on moribund or dead fish and transmit infection from one pond or cage to another. These birds are also involved in the transmission of digenean parasites, *Francisella* spp., *Edwardsiella tarda*, and viral pathogens [43]. In this study, the most common methods of controlling these birds include the use of bird nets and physical chasing, though these are not very effective as birds could still have access to fish.

As with many research activities, limitations were present. In our case, time and resources (financial) were among the limiting factors. A longitudinal study was going to be suitable instead of the cross-sectional study done. It would account for environmental changes (temperature, pH) and seasons that affect the microbiota in the water where fish live and the microbiota of fish. Failure to identify some of these bacteria isolates could be attributed to gaps in the diagnostic capability of the techniques used, and this could be a group of bacteria representing new genera or species in the fish samples we collected. More time and financial resources are needed. Sampling and analysis biases were the two forms of biases encountered in this study. To lower the sampling bias, three different farms were involved in the sampling process. Sampling was spread across the different production systems (cage and pond systems), and it was nonprobabilistic; hence, our analysis did not involve the use of more statistical tools, thereby reducing the level of biases in this study.

In conclusion, given its limitations and strengths, this current study has been able to provide baseline information with regard to future reference as well as being an important stepping stone with regard to the implementation of public health guidelines which have potential zoonotic implications arising from fish.

## Figures and Tables

**Table 1:** Exposure factor locations identified from on-farm visitations and observations.

Demographic factors	Biological factors	Environmental factors	Management factors
Hatchery	Hatchery	River	Hatchery
Sex reversal pond	Breeding stock	Fixation pond	Sex reversal pond
Breeding stock	Male pond	Sex reversal pond	Out-grower pond
Male pond	Out-grower pond	Empty pond	Fingerling pond
Out-grower pond	Andersonii pond	Draining pond	Rejects pod
Andersonii pond	Niloticus pond	Stabilization pond	Juvenile cage
Rejects pond	Machrochir pond	Out-grower pond	
Machrochir pond	Mixed pond fingerling pond	Out-grower cage	
Niloticus pond	Out-grower cage	Lake	
Fingerling pond	Juvenile cage	Hatchery outlet	
Out-grower cage			
Juvenile cage			

**Table 2:** Occurrence of the number of bacteria isolated from fish verses water per farm.

Bacteria genera	Occurrence *n* (%)						Overall (*n* = 153)
	Farm A (*n* = 60)		Farm B (*n* = 51)		Farm C (*n* = 42)		
	Fish	Water	Fish	Water	Fish	Water	
*Acinetobacter*	0	2	1	0	0	0	3 (2.0)
*Aequorivita*	0	2	0	0	0	0	2 (1.3)
*Aerococcus*	0	0	0	0	2	0	2 (1.3)
*Aeromonas*	8	10	1	0	0	0	19 (12.4)
*Bacillus*	0	1	0	1	1	0	3 (2.0)
*Bordetella*	0	0	3	0	0	0	3 (2.0)
*Carnobacterium*	1	2	4	3	3	2	15 (9.8)
*Citrobacter*	0	4	0	0	1	0	5 (3.3)
*Clostridium*	2	0	0	0	1	0	3 (2.0)
*Corynebacterium*	0	0	1	0	1	0	2 (1.3)
*Dermatophilus*	0	0	1	0	1	0	2 (1.3)
*Enterococcus*	0	1	2	0	0	0	3 (2.0)
*Escherichia coli*	0	1	0	0	0	0	1 (0.7)
*Flavobacterium*	1	2	2	0	0	1	6 (3.90
*Klebsiella*	5	5	0	0	0	0	10 (6.5)
*Lactococcus*	0	0	3	0	5	0	8 (5.2)
*Lactococcus/Streptococcus*	0	0	1	0	0	0	1 (0.7)
*Listeria*	1	0	0	0	0	0	1 (0.7)
*Micrococcus*	0	0	5	2	2	1	10 (6.5)
*Planococcus*	0	0	1	0	0	1	2 (1.3)
*Proteus*	0	2	0	0	0	0	2 (1.3)
*Pseudomonas*	0	0	8	0	0	1	9 (5.9)
*Rhodococcus*	1	0	1	0	0	0	2 (1.3)
*Shewanella*	0	0	1	0	0	1	2 (1.3)
*Staphylococcus*	3	6	2	2	2	11	26 (17.0)
*Streptococcus*	0	0	1	0	0	0	1 (0.7)
*Vagococcus*	0	0	0	0	1	0	1 (0.7)
Unidentified bacteria	0	0	3	2	2	2	9 (5.9)

The tables show the number of isolated bacteria from fish verses water per farm.

**Table 3:** Biochemical characteristics of bacterial isolates isolated from tilapia (*Oreochromis niloticus*) and water samples from farms A, B, and C.

Bacterial isolates	Tests conducted																										
	GM	CM	H	Ox	Cat	Esc	Gal	Raf	Sal	Mal	Xyl	Man	Tre	Inu	Sor	Lac	Urea	Glu	Suc	lac	g	H_2_S	Cit	sul	mot	Ind	MR
*Acinetobacter* spp.	−	Rods	*β*	+	+	−	−	−	−	−	−	−	−	−	−	−	−	−	−	−	−	−	+	−	−	−	−
*Aequorivita* spp.	−	Rods				−	−	−	−	−	−	−	−	−	−	−	−	+	−	−	−	−	+	−	−	−	−
*Aerococcus* spp.	+	Cocci	*α*		+	+	+	+	−	+	+	+	+	−	+	+	+	+	+	+	−	−	−	−	+	−	−
*Aeromonas* spp.	−	Rods	*β*	+	+	−	+	−	−	+	−	−	+	−	−	−	−	+	+	+	−	−	−	−	+	+	−
*Bacillus* spp.	+	Rods	*β*		+	−	−	−	−	−	−	−	−	−	−	−	−	−	−	−	−	−	+	−	+	−	−
*Bordetella* spp.	−	Rods		+	+	−	—	−	−	−	−	−	−	−	−	−	+	+	−	−	−	+	+	−	+	−	−
*Carnobacterium* spp.	+	Rods	*α*	−	−	−	−	−	−	−	−	−	−	−	−	−	−	−	−	−	−	−	−	−	−	−	−
*Citrobacter* spp.	−	Rods		−	+	−	−	−	−	−	−	−	−	−	−	−	+	−	−	−	−	−	+	−	+	−	+
*Clostridium* spp.	+	Cocci			−	−	−	−	−	−	−	−	−	−	−	−	−	−	−	−	−	−	+	−	−	−	+
*Corynebacterium* spp.	+	Rods	*β*	−	+	−	−	−	−	−	−	−	−	−	−	−	+	−	−	−	−	−	−	−	−	−	−
*Dermatophilus* spp.	+	Cocci	*β*		+	−	−	−	−	−	−	−	−	−	−	−	+	+	−	−	−	−	+	−	+	−	−
*Enterococcus* spp.	+	Cocci		−	−	−	−	−	−	−	−	−	−	−	−	+	−	+	−	−	−	−	+	−	−	−	−
*Escherichia coli* spp.	−	Rods		−	+	+	+	+	+	+	+	+	+	−	−	+	−	+	+	+	−	−	−	−	+	−	+
*Flavobacterium* spp.	−	Rods		+	+	−	+	−	−	+	−	−	+	−	−	+	−	+	+	+	+	−	−	−	+	−	−
*Klebsiella* spp.	+	Rods				−	+	+	−	+	+	+	+	−	+	+	−	+	+	+	+	+	+	+	+	+	−
*Lactococcus* spp.	+	Cocci	*α*	−	−	−	+	−	−	−	−	−	−	−	−	−	+	+	+	+	−	−	−	−	+	−	+
*Lactococcus/Streptococcus* spp.	+	Cocci		−	−	−	−	−	−	+	−	−	−	−	−	−	−	+	+	+	−	−	−	−	+	−	+
*Listeria* spp.	+	Rods				−	+	−	−	+	−	+	+	−	−	−	−	+	+	+	+	−	+	−	+	+	−
*Micrococcus* spp.	+	Cocci		+	+	−	−	−	−	−	−	−	−	−	−	−	−	−	−	−	−	−	−	−	−	−	−
*Planococcus* spp.	+	Cocci		−	+	−	−	−	−	−	−	−	−	−	−	−	+	+	+	+	−	−	−	−	+	−	−
*Proteus* spp.	−	Rods		−	+	+	+	−	+	+	−	+	+	−	−	−	−	+	+	+	−	−	−	−	+	+	−
*Pseudomonas* spp.	−	Rods		+	+	−	−	−	−	−	−	−	−	−	−	−	−	−	−	−	−	−	+	−	+	−	−
*Rhodococcus* spp.	+	Rods		−	−	−	−	−	−	−	+	−	−	−	−	−	+	−	−	−	−	−	−	−	−	−	−
*Shewanella* spp.	−	Rods	*β*	+	+	−	−	−	−	−	−	−	−	−	−	−	−	+	+	+	−	−	+	−	+	−	−
*Staphylococcus* spp.	+	Cocci	*β*	−	+	−	+	−	−	+	+	+	+	−	+	−	−	+	+	+	+	−	−	−	−	+	−
*Streptococcus* spp.	+	Cocci	*β*	−	−	−	−	−	−	+	−	−	+	−	−	−	−	+	+	+	−	−	−	−	−	−	−
*Vagococcus* spp.	+	Cocci	*α*	−	−	−	+	−	−	−	+	−	−	−	−	−	−	+	−	−	−	−	+	−	−	−	−
*Unidentified 1*	+	Cocci				+	+	−	−	−	−	−	−	−	−	−	−	+	+	+	−	−	+	−	+	−	+
*Unidentified 2*	+	Rods				−	+	−	−	+	−	−	+	−	+	+	−	+	+	+	−	−	+	−	+	−	+
*Unidentified 3*	+	Cocci				+	+	+	−	+	−	−	+	−	+	−	+	+	+	+	−	−	+	−	+	−	-
*Unidentified 4*	−	Rods				+	−	−	−	−	−	−	−	−	−	−	−	−	−	−	−	−	−	−	+	+	-
*Unidentified 5*	+	Cocci				−	−	−	−	+	−	−	−	−	−	−	−	−	−	−	−	−	−	−	−	−	-
*Unidentified 6*	+	Cocci				−	−	+	−	+	−	−	+	−	+	−	+	+	+	+	−	−	−	−	+	−	+
*Unidentified 7*	−	Cocci				−	−	−	−	−	−	−	−	−	−	−	+	−	−	−	−	−	−	−	+	−	+
*Unidentified 8*	+	Rods				−	−	−	−	+	−	+	−	−	−	−	−	+	+	+	−	−	−	−	−	−	-
*Unidentified 9*	+	Cocci				−	−	−	−	−	−	−	−	−	−	−	−	+	−	+	−	−	−	−	−	−	-

GM = Gram stain, CM = cell morphology, H = hemolysis, Cat = catalase, Ox = oxidase, Esc = esculin, Gal = galactose, Raf = raffinose, Sal = salicin, Mal = maltose monohydrate, Xyl = xylose, Man = mannitol, Tre = trehalose, Inu = inulin, Sor = sorbitol, Lac = lactose monohydrate, Urea = urease, Glu = glucose, Suc = = sucrose, lac = lactose, g = gas production, H_2_S = production of hydrogen sulphide, Cit = citrate, Sul = sulphur, Mot = motility, Ind = indole, and MR = methyl red.

**Table 4:** Occurrence of the different bacteria genera on farms A, B, and C.

Bacteria genera	Occurrence *n* (%)			Overall (*n* = 153)
	Farm A (*n* = 60)	Farm B (*n* = 51)	Farm C (*n* = 42)	
*Acinetobacter*	2 (3.3)	1 (2.0)	0 (0.00)	3 (2.0)
*Aequorivita*	2 (3.3)	0 (0.00)	0 (0.00)	2 (1.3)
*Aerococcus*	0 (0.00)	0 (0.00)	2 (4.8)	2 (1.3)
*Aeromonas*	18 (30)	1 (2.0)	0 (0.00)	19 (12.4)
*Bacillus*	1 (1.7)	1 (2.0)	1 (2.4)	3 (2.0)
*Bordetella*	0 (0.00)	3 (5.9)	0 (0.00)	3 (2.0)
*Carnobacterium*	3 (5.0)	7 (13.7)	5 (11.9)	15 (9.8)
*Citrobacter*	4 (6.7)	0 (0.00)	1 (2.4)	5 (3.3)
*Clostridium*	2 (3.3)	0 (0.00)	1 (2.4)	3 (2.0)
*Corynebacterium*	0 (0.00)	1 (2.0)	1 (2.4)	2 (1.3)
*Dermatophilus*	0 (0.00)	1 (2.0)	1 (2.4)	2 (1.3)
*Enterococcus*	1 (1.7)	2 (3.9)	0 (0.00)	3 (2.0)
*Escherichia coli*	1 (1.7)	0 (0.00)	0 (0.00)	1 (0.7)
*Flavobacterium*	3 (5.0)	2 (3.9)	1 (2.4)	6 (3.90
*Klebsiella*	10 (16.7)	0 (0.00)	0 (0.00)	10 (6.5)
*Lactococcus*	0 (0.00)	3 (5.9)	5 (11.9)	8 (5.2)
*Lactococcus/Streptococcus*	0 (0.00)	1 (2.0)	0 (0.00)	1 (0.7)
*Listeria*	1 (1.7)	0 (0.00)	0 (0.00)	1 (0.7)
*Micrococcus*	0 (0.00)	7 (13.7)	3 (7.1)	10 (6.5)
*Planococcus*	0 (0.00)	1 (2.0)	1 (2.4)	2 (1.3)
*Proteus*	2 (3.3)	0 (0.00)	0 (0.00)	2 (1.3)
*Pseudomonas*	0 (0.00)	8 (15.7)	1 (2.4)	9 (5.9)
*Rhodococcus*	1 (1.7)	1 (2.0)	0 (0.00)	2 (1.3)
*Shewanella*	0 (0.00)	1 (2.0)	1 (2.4)	2 (1.3)
*Staphylococcus*	9 (15.0)	4 (7.8)	13 (31.0)	26 (17.0)
*Streptococcus*	0 (0.00)	1 (2.0)	0 (0.00)	1 (0.7)
*Vagococcus*	0 (0.00)	0 (0.00)	1 (2.4)	1 (0.7)
Unidentified bacteria	0 (0.00)	5 (9.8)	4 (9.5)	9 (5.9)

The table shows occurrence of the different bacteria genera isolated from the three farms.

## Data Availability

All data used for the study are available upon request from the corresponding author.
